# Subscriptions from Members of the College

**Published:** 1920-10-30

**Authors:** 


### Subscriptions from Members of the College.

Long before the last annual meeting of the College of Nursing it had become clear that the Council could not much longer continue to offer nurses admission on the easy terms of a guinea fee to include registration and life membership. The present number of members, which now approaches the 20,000 for which we pleaded at the outset, promises unchecked growth. Its influence increases day by day in proportion to its numbers. The advantages it offers to its members, the prestige it is able to afford, and its position as a national institution abundantly justify the new departure forecasted, under which membership is to carry with it the duty and privilege of subscribing annually to its support. At the beginning, when the purpose was to rally the entire profession and bring them under one banner, we heartily approved the policy of admitting to membership and registration on payment of a small inclusive fee. But a hundred new directions have opened up in which development may be pushed. The expenses of running societies has enormously increased. Salaries, postage, printing, travelling have doubled. More is expected, much more can be given, than was dreamed of in 1916. The hour for imposing an annual subscription has come, and there is in the ranks of College members, so far from any recoil from this duty, the fact that many have long voluntarily discharged it. Apart from other considerations, all admit that those who join from now on stand in a very different position compared with those who joined in faith in the early days.

The admirable organising ability displayed at headquarters has succeeded in meeting the difficulty inherent in dealing with large numbers of nurses, many of whom have no fixed address. Of the members elected each year a certain proportion automatically fail to make good as time goes on. From various causes—emigration, marriage, change of career, death itself—some 10 per cent., it may be predicated, will always fall away. Perhaps another 5 per cent. fall away from laziness. Unless the College has power to deal with defaulters these "dead" members clog the wheels. The advantage of a fixed subscription is seen in the power which it gives to distinguish the live members and to keep a strong, living current stirring through the College.

In course of time all will be subscribing members. Even if the subscription is fixed at no more than 5s., an annual revenue of some £250 from every 1,000 members will carry the College some way on the path of progress.

